# Healthcare Management: A Bibliometric Analysis Based on the Citations of Research Articles Published between 1967 and 2020

**DOI:** 10.3390/healthcare10030555

**Published:** 2022-03-16

**Authors:** Oana Păduraru, Alina Moroșanu, Călin Ștefan Păduraru, Elena Mihaela Cărăușu

**Affiliations:** 1 Doctoral School, Grigore T. Popa University of Medicine and Pharmacy, 700115 Iasi, Romania; oanaforest71@yahoo.com; 2 Department of Social Sciences and Humanities, Institute of Interdisciplinary Research, Alexandru Ioan Cuza University, 700506 Iasi, Romania; 3 Doctoral School of Biomedical Sciences, Dunărea de Jos University, 800008 Galati, Romania; calinstefanpaduraru@gmail.com; 4 Department of Public Health and Management, Grigore T. Popa University of Medicine and Pharmacy, 700115 Iasi, Romania; mihaelacarausu@yahoo.com

**Keywords:** healthcare, health management, bibliometric analysis

## Abstract

The purpose of this study is to analyse the trends manifested in research literature from the field of healthcare management, with emphasis on bibliometric features and different influencing factors. For this, a search was conducted of nine academic databases between January and May 2021. Article features were registered in our database after first applying the validation criteria used for their inclusion. Then, data regarding the publication of the included articles were collected. The analysis focused on trends over time, topic, and journals in which they were published. Moreover, the effect of some factors on the citation of articles was analysed. Our results showed that the 250 analysed articles were published in 139 journals, and many of were by researchers affiliated with universities in the United States. Over time, the publication of analysed articles and their number of citations registered a continuous increase. The most common topics of focus were healthcare management systems and their challenges. In our study, we identified factors that significantly affect citation number, such as number of years since publication, the number of words in the title, and the number of authors of an article. In addition, major gaps were identified, as were new unresolved challenges that can trigger new research ideas.

## 1. Introduction

In recent years, there has been constant concern in the field of healthcare, considering the risks to the health of the population caused by severe pandemics, the most recent example being the COVID-19 pandemic [1,2]. The COVID-19 pandemic has shown more than ever that the most valuable source for generating innovation in the healthcare management field is concern and not only information. To increase the chances of generating innovations, the amount of useful information must also increase [3]. Evidence of this is the provision and rapid sharing of scientific data and information. Since the beginning of the pandemic on platforms such as GISAID or Nextstrain, the amount of data shared has increased significantly. Moreover, 117 organisations (including journals, funding agencies, disease prevention centres) signed a statement entitled “Sharing relevant data and research findings for the new coronavirus outbreak (COVID-19)” [3]. Thus, a series of practices related to the elimination of payment for accessing scientific documents and the use of preprint servers were initiated [4]. Researchers and practitioners from all over the world have continued this initiative, encouraging people to make their work available to help in fighting the COVID-19 pandemic [3]. Programs, such as CORD-19 (CORD-19, 2020), MOBS Lab, MIDAS, ELIXIR, COVID-19 Data Portal, or COVID-19 High-Performance Computing Consortium, can provide a variety of resources as well for scientific research [4]. In addition to sharing data and research tools, the rapid dissemination of research results played an important role in building an objective dialogue that helped to facilitate the process of generating new research directions [3].

Thus, researchers and academia have a key role to play in developing and promoting the highest level of healthcare [5,6]. The results of research in several fields contribute significantly to the identification and adoption of important solutions that help to achieve future objectives. Many of the organisations that provide healthcare services face the existence of quite serious problems that can be solved through research and innovation [5]. In other words, researchers are best placed to propose genuine innovative solutions that actively contribute to solving national and international problems [5]. Given this context, local and international authorities are free to implement research results and academic recommendations. They could provide a practical agenda that would strengthen the partnership with all stakeholders and help to accelerate the steps towards its fulfilment. On the other hand, the plurivalence of the research field is a way of understanding the fact that there is a non-linearity, and behaviours are the factors that determine the set rules [7]. They have developed through academic customs in the fields of exact sciences and humanities. The diversity and complexity of systems can be understood by identifying common traits and behavioural patterns that are based on distinct units [8]. In the areas related to health and healthcare, this diversity is provided by the multitude of involved categories (health professionals, health authorities, beneficiaries, non-governmental organisations) and the interactions between them [9]. This approach was initiated with the dissemination and publication of the results of studies and research debating the application of scientific plurivalence in the healthcare [10,11,12] or education [13] domains.

Global warming also presents a risk to the population through the rapid transmission of infectious diseases [11]. Conflicts in different geographical areas of the world have also led to the mass migration of refugees, with this category of people benefiting from minimal healthcare services [12,13]. Lack of well-being, cleanliness, access to medicines and healthcare services has increased the risk of spreading diseases to the most exposed categories of the population (children, women, and the elderly) [13]. The global onset of health challenges, in general, and healthcare imposed by spontaneous and unpredictable pandemics, conflicts, climate change, and the lack of economic growth have impacted welfare and the fulfilment of development goals.

Taking in consideration this context, the ability of research to understand and address some of the biggest challenges in healthcare delivery was observed. An edifying example is provided by [14], in which complexity theory is used to highlight and analyse how nurses in the United States make decisions. Another team of researchers [15] conducted a case study evaluating how decisions were made regarding the financing of hospitals in Kenya.

Therefore, in the specialised literature, there are indications according to which the study of the field of healthcare reaches a certain depth. They come as a complement to what is already known, and an examination of the influence and extent of this literature is even more beneficial. Bibliometric analysis offers an objective image of the publications from a certain area of the specialised literature. On the other hand, as specified above, bibliometric analyses cannot escape the intellectual properties contexts whereby researchers and policymakers (and funders) strive for innovative solutions in healthcare management and beyond.

### Background and Objectives

Bibliometric analysis can be used to identify many papers from the recent literature that provide a general overview regarding different aspects of healthcare management. For example, some authors [16] have presented a view of healthcare-related research and the directions of future work to benefit patients and healthcare providers. Others [16,17,18,19] highlight dynamic trends in publications and have identified the most influential authors, institutions, countries, and research teams for a certain journal case. However, those studies are limited to broad directions on the development of healthcare. Another limitation they have is in regard to the definitions of the healthcare management field. Because some additional terms (for example, “sustainability” or “artificial intelligence”) were used to identify papers that treat aspects of healthcare management, there was a high risk of including sample documents on topics related to other fields, such as innovation, environment, or sociology. For this reason, we consider it necessary to have a clearer definition of healthcare management. In addition, a limitation of some papers that present a bibliometric analysis of healthcare management is the data source used [20,21]. An article search can be performed using other additional databases (for example, Scopus or Google Scholar), as suggested in another study [22].

Given these limitations, which we found by consulting the research literature, the aim of our paper is to perform a bibliometric analysis of the literature dealing with healthcare management by analysing the effect of different factors such that other researchers can then use the results in further studies. Moreover, another part of our goal is to identify aspects of healthcare management research that require further attention from researchers. This would serve to encourage future research in the areas of need, which would benefit this field of research.

In achieving this goal, we attempt to provide answers to the following questions:

▪ Is there variability between journals regarding the number of citations received by academic articles?
▪ Does the number of citations of an article vary depending on the journal in which it is published?
▪ Does the number of citations of an article vary depending on its topic?
▪ Are discrepancies in the citations of articles between areas due to dissimilarities in the level of developed research infrastructure?

Taking into consideration the proposed goal and the research questions, the following research objectives were established:

O1: Analysis of the dynamics of scientific “production” related to healthcare management field.
O2: Highlighting scientific progress as well as identifying the most prolific researchers, institutions, and countries in which research in this field is carried out [23].
O3: The study of the influence of some factors upon which the citations of the articles dealing with this topic are dependent.

## 2. Materials and Methods

### 2.1. International Literature Search

#### 2.1.1. Databases

To identify articles dealing with the topic of healthcare management, international databases, such as JAMA Network, JSTOR, PMC, PubMed, SAGE, ScienceDirect, Springer, Taylor & Francis, and Clarivate, were consulted. This activity took place between January and May 2021. Our approach involved the use of keywords that relate to healthcare management. No limits have been imposed for identifying articles, research reports, reviews, or books. In addition, the number of citations was used in cases where it was necessary to identify papers of high scientific relevance. In total, by searching the nine international databases, 1594 articles were identified. They were downloaded into an Excel spreadsheet, and duplicates were then removed. After this stage, 943 articles remained, and their details were saved in another spreadsheet in Excel with the purpose being to examine them so that only those titles that met the requirements specified above are retained for analysis. For those 943 articles, the titles/abstracts were checked, and 651 of them were excluded on the basis that their content was strongly related to other fields. We wanted to ensure that we would only consider articles that are not related to other fields for analysis and that there were no other compelling reasons for exclusion. Thus, we obtained 292 articles for which another evaluation was conducted, and other reasons for exclusion were identified in the case of another 42 articles (Table 1).

After this stage, 250 articles were retained and subjected to analysis, although another 1344 had already been excluded in earlier stages because they did not meet the inclusion criteria during verification.

**Figure 1:**
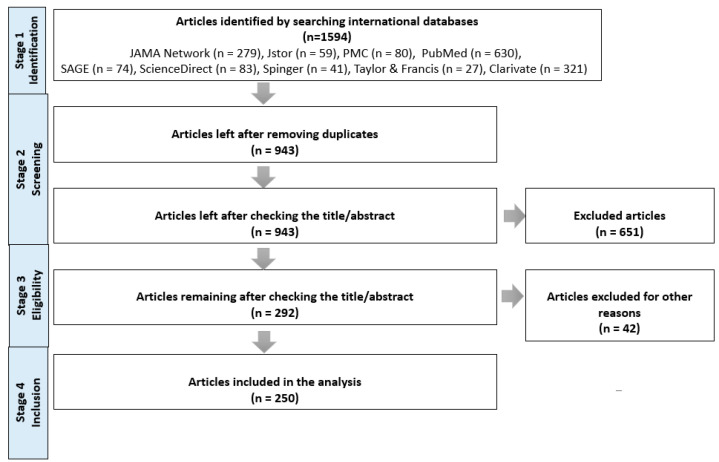
Research strategy applied for the selection of articles included in analysis.

Of the 250 articles considered eligible for analysis, the full text in English was accessed for 223 articles, thus making it possible to achieve a more detailed and clear classification of the content. In the case of an additional 20 articles that were included in the bibliometric analysis and were published in journals in English, their text was only partially accessible, and in the case of the final 7 articles, the text was not available in English. Figure 1 presents a summary of the details concerning the search strategy.

#### 2.1.2. Keywords

To identify the most appropriate keywords to use in identifying articles dealing with healthcare management topic, it was necessary to first define this concept. Thus, in some sources [24], healthcare management is defined as “the profession that provide leadership and direction to organizations that deliver personal health services, and to divisions, departments, units, or services within those organisations”. Based on this definition, we can consider that healthcare management involves the planning and coordination of nonclinical activities within healthcare systems, organisations, and networks.

Database searching was conducted using “healthcare management” OR “healthcare hospital” OR “health system” OR “primary care” OR “clinical management” OR “acute care” OR “healthcare practice” OR “medical practice” OR “healthcare networks” OR “nursing management”. Those keywords must occur in the article’s title.

References identified using these keywords were managed using EndNote. Thus, duplicates were much more easily found and then removed from our list. All references that met our criteria were retained for analysis. In the next step, all data were exported to an Excel spreadsheet in which variables represent each of the researched aspects were created. For example, to quantify the number of authors of an article, the variable “Author” was created. To include an article in a certain category that designates the research area, the variable “Topic” was created. To identify the journal in which the article was published, the variable “Journal” was created, or to see the country from which most articles came, the variable “Country” was created. After the database was created, the statistical program R was used to obtain the results.

#### 2.1.3. Criteria Used to Include Articles in the Analysis

The analysis will include articles published in academic journals with impact factors indexed in international databases and referring to healthcare management. It was also intended that these articles include results obtained not only by specialists who provide medical assistance (nurses, doctors) or specialised websites of institutions (such as hospitals, retirement homes) but also people specialised in other fields related to health (for example, education, ethics, policies, research).

Articles that, through their published results, promote aspects of reference to healthcare management, in general, were initially included in our archive. Following this action, some were removed because it was later found that they did not refer to healthcare and care platforms, the profession, or issues related to the provision of medical care services. Those articles referring to interventions or health promotion issues carried out at the population level were not included.

Editorial works, books, chapters, and articles published in volumes of certain correlations were also excluded because it was considered that the information regarding them was either not available or incomplete. This includes articles that, although initially not published in English, have a summary in English based on which it can be verified that they meet our criteria.

### 2.2. Bibliometric Analysis

#### 2.2.1. Searching Bibliometric Data

The bibliometric data (year of publication, number of authors, the journal in which it was published, the country of residence of the main author) of each article were recorded from the text of the paper, from the journal that published the article, or from the database in which the journal was indexed. In some cases, it was found that the full text was not available, was inaccessible to the public in its entirety, or was not written in English.

#### 2.2.2. Articles Content Evaluation

At this stage, the articles were grouped together, considering their content, the type, and the topic. This involved classifying articles as empirical or non-empirical. The articles that, through the published results, refer to a certain conceptual framework, methods of data analysis, or ways of interpreting some results were considered empirical. Included in the category of non-empirical works were case studies, reviews, editorials, and debates on conceptual criteria.

Another important aspect refers to the analysis of the content of the registered articles. Thus, a code was assigned to each article and comparisons were conducted, and changes were then made to the codes to identify the most frequently encountered research topics. These details were identified from the title of the articles or their abstract. These were also identified for articles in which the summary did not provide sufficient detail. In those cases, the full verification of the article content was carried out where the text was available in English.

#### 2.2.3. Determination of Articles Influence

To determine the influence that an article has in the literature of the healthcare management field, the number of citations received over time was considered. For this purpose, Google Scholar was consulted. In addition, the references of each article whose text could be accessed and viewed in full version were considered. An essential condition was that the reference should not be made by a publication evaluated by fellow authors, which allowed ensuring greater objectivity in terms of considering other types of academic results. It is also important to mention that no patients or other categories of the population were involved in this research.

### 2.3. Evaluation of Article Influence

It is known that the number of citations received by an article is associated with his scientific quality. The impact factor (IF), Hirsch index (H index), crown indicator, and other appropriate metrics that take the citation number into consideration were calculated. In addition, scientific search engines used for finding references (such as Google Scholar) show the list of results according to the number of citations [25]. For these reasons, the number of citations that a scientific paper in the field of healthcare management receives is an important element used to assess the impact of research. However, the number of citations of an article may be influenced by different factors. According to [26,27,28], the number of citations of an article is affected by factors such as their length, author number, number of institutions at which the authors are affiliated, number of words in the title, number of cited references, topic, or time since publication.

Articles length (the number of pages) could be a factor that can significantly affect the citations received. A large article can reflect a greater scientific complexity and a higher quality of used methodology; in addition, a lengthier article is expected to contain a greater amount of information, increasing the possibility that it can be appropriated to be cited by other researchers [29]. In other words, in articles with a larger number of pages compared to those with a smaller number, the research methodology and the obtained results could be presented and discussed more clearly and in detail. Therefore, their impact on citation number could be higher.

The number of authors attracts citation because it is considered that the higher number of authors of an article can determine more traffic for the paper, and so the chances of obtaining more citation are increased [30,31]. On the other hand, articles that cite more references and more publications referenced in different research platforms get a fairly large number of citations.

The number of institutions at which the authors of an article are affiliated is also important. Multiple affiliation is increasingly considered as stimulating knowledge exchange. An individual scientist may seek affiliations to obtain access to research resources, research infrastructure, or career opportunities. Co-authorship can be an efficient way of developing the competencies of a research group, and co-affiliation may be a way of forming stronger connections between researchers and institutions [32].

Articles title length is perceived as having an important role in the strategy of attracting audience and citations [33]. Longer titles contain more words and, therefore, more potential keywords, increasing the chances of an article being found easier. On the other hand, longer titles may be harder to digest and thus less attractive [34].

The number of references in healthcare management articles is another factor that can affect the citation. The reason may be related to the reason researchers from this field cite other’s work. From a theoretical perspective, a reference list of high quality is a comprehensive and well-balanced selection of papers, which can support the content presented in a certain article. Researchers usually reach this by performing a retrospective search and selection of papers with content that is pertinent to that of their article, to be read and cited. Well-chosen and mentioned bibliographic references can support the novelty, value, and visibility of an article. Citations can link a study to other studies, thus creating a network of knowledge that allows other researchers to identify studies that are relevant in general and relevant to them.

Commonly, articles published recently have a limited number of citations, an increase being seen after 3 or 5 years since publication [35]. Therefore, when analysing citation frequencies, the temporal dimension is important too.

Furthermore, the nationality of the authors is important. This can show that a specific research culture that is associated with a certain geographical area can influence the progress of research in a certain field and the number of citations received.

Giving this context, we want to consider those factors identified in the research literature and to verify if those results are also the same in our case. Therefore, one of our research objectives is to identify the factors associated with obtaining citations so that we can explain their effect and offer useful suggestions for increasing citations. To do this, the following variables will be considered for analysis: journal name, number of title words, number of authors, time since each article was published, the paper length (pages number), the number of references in each article, and the country of the affiliated institution of the first author. The characteristics of those variables are presented in Table 2.

#### Data Analysis Methods

A multilevel regression model was used to study the influence of factors, such as article author number, article topic, researcher affiliation, or the number of words in the title, on the number of citations of the considered articles. Multilevel models describe hierarchical structures referring to quantifications taken on the same unit at various moments, e.g., physical characteristics that are strongly correlated compared to previous assessments from different units. Multilevel models are used for the analysis of such dependency.

Multilevel modelling allows us to analyse the type of between-group variability and the outcomes of a group-level attribute on single outcomes. To offer an answer to our research questions, we opted for a two-level pattern with articles at level 1, nested with groups at level 2. Moreover, in a two-level model, residuals are divided into two parts, suitable for the two levels in the data frame. The random effects (group-level residuals) are noted by *u_i_* and the individual residuals group is noted by *e_ij_.* The two-level extensions that allow for group effects are given by

(1) $$Y_{ij}=\beta_{0}+u_{j}+e_{ij}$$

where $\beta_{0}$ is the gross average of *Y* (through all groups). The average of *Y* for group *j* is $\beta_{0}+u_{j}$. The residual $u_{j}$ is the difference between the average of group *j* and of the total. The individual-level residual $e_{ij}$ is the difference between the *Y* value for the *i* unit and that unit’s mean group $e_{ij}=Y_{ij}-\left(\beta_{0}+u_{j}\right)$. Residuals for both levels are assumed to be normally distributed with 0 means: $u_{j}\sim N(0,\sigma_{u}^{2}$) and $e_{ij}\sim N\left(0,\sigma_{e}^{2}\right)$. The simplest multilevel model with one explanatory variable is

(2) $$Y_{ij}=\beta_{0}+\beta_{1}X1_{ij}\ldots \beta_{n}Xn_{ij}+u_{j}+e_{ij}$$

In this model, the relationship between *Y* and *X* is expressed by a direct line with intercept $\beta_{0}$ and slope $\beta_{1}$.

## 3. Results

### 3.1. Results Regarding the Dynamic of Scientific Production Related to the Healthcare Management Field

The 250 articles included in the analysis come from 139 different journals in the field of healthcare management, and they have a varied impact. Table 3 presents the journals in which the most articles in this field were published.

The results show that the journal in which the highest number of articles in the field of healthcare management was published is JAMA (n = 34), followed by Health System in Transition (n = 11) and Libyan Journal of Medicine (n = 7).

**Figure 2:**
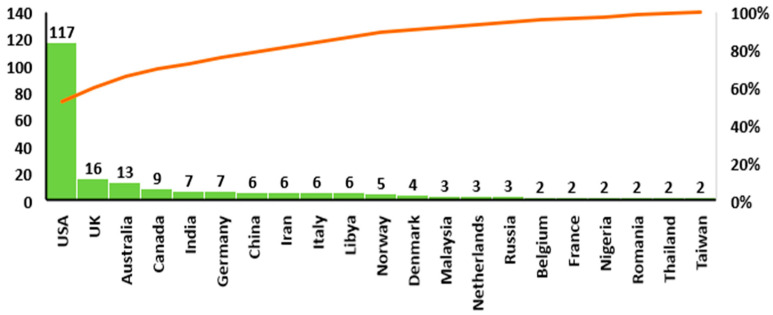
Distribution of published articles in the domain of healthcare management worldwide.

Regarding the affiliation of the main author, it was observed that the analysed articles are from 48 countries. The United States 47% (n = 117), the United Kingdom 6% (n = 16), Australia 0.5% (n = 13), and Canada 4% (n = 9) together accounted for 62% of the total items included in the analysis, as can be seen in Figure 2. Other countries with a higher contribution are India, China, Iran, Italy, and Libya (all with six published articles. each with a contribution of 2%).

**Figure 3:**
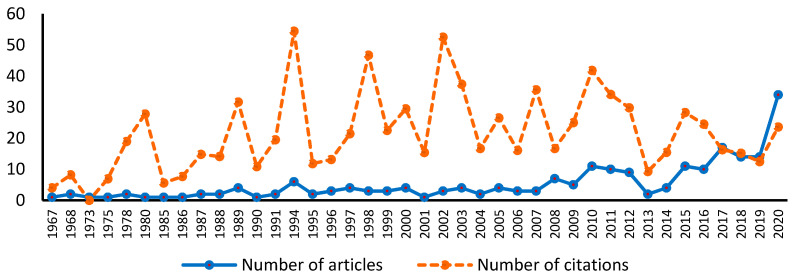
The number of articles and number of citations according to year.

Although most of the articles have been published by authors who are affiliated with universities in only a few countries, the results show that there has been significant growth in recent years, especially in the early period of the pandemic (Figure 3). This result can show an increasing speed regarding the dissemination of research findings, many journals prioritising the processing of articles involving COVID-19. Related to this aspect, an analysis of a limited number of journals showed that the number of days between an article submission and article publication during the pandemic was decreased by almost half compared to that before the pandemic.

Before 2000, the only countries that published literature on healthcare management were the United States, the United Kingdom, or Germany; since 2015, the articles have come from 19 different countries. The published articles also come from authors affiliated with universities from Finland, Norway, the Netherlands, Denmark, Spain, Switzerland, Romania, China, the Philippines, Pakistan, and Nigeria.

### 3.2. Results Regarding Articles Topic

All articles included in the analysis could be classified with full and accessible text. Around 12.4% of them (*n* = 31) treated the conceptual elements regarding healthcare management as well as elements of “literature review” (Table 4).

Around 44.5% of the articles deal with management systems in terms of healthcare and the encountered challenges and the quality of these services. Both topics are addressed mainly in works by researchers from the United States (n = 46), Canada (n = 7), Australia (n = 5), and the United Kingdom (n = 4).

The results of such studies were obtained by large groups of researchers, with the number of authors of the articles exceeding 10 (in the case of articles published by authors in the United States or Canada). This result shows that there are many researchers who also contributed to the article, and these results are often being obtained because of large research projects that require multidisciplinary teams.

Moreover, as the field of healthcare management develops, its magnitude is reflected in the number of authors who publish their scientific papers. Lately, more and more often, the research is based on the efforts of larger groups of researchers. There is also a disadvantage of having many authors since there have been cases in which the article cannot be reviewed because almost all the experts in a certain field were among its authors.

Another important aspect highlighted by the analysed articles is the fact that in the last 2 years, the number of articles that were published in the domain of healthcare management has greatly increased. Most of the articles published in these 2 years refer to healthcare management systems and their challenges, the quality of these services, or access to them.

This result is not accidental, as more and more countries are facing many problems caused by the COVID-19 pandemic. For decisions to be made about healthcare for individual patients and public health policies, the best confirmations available based on research are needed. This creates the framework for ensuring best practices and reducing variations in the provision of healthcare. For this reason, the large number of articles is justified, requiring the latest and most reliable information.

Going deeper into the content of the selected articles, it was possible to develop broader categories in terms of the addressed topic, with several topics being frequently prominent. These topics are listed in Table 3 and refer to “Characteristics of health systems in healthcare management”, “Impact of factors on healthcare management”, “Healthcare legislation”, or “Good management practices”.

Although health professionals have addressed various topics dealing with healthcare management since 1967, it was only in 2015 that articles began to focus on change, growth, and implementation, research, and policy.

### 3.3. Influential Articles and the Effect of Factors on Which the Citation of Analysed Articles Depend

The number of citations of an article in a certain period, regardless of the field, can show the importance and appreciation given to the results obtained by members of the medical, scientific community.

The number of citations of each article was used to highlight the influence of the analysed articles. In total, the average number of citations is 101, although the degree of asymmetry is high (median = 20.5, min. = 3, max. = 1752).

The most influential article in our database was published in Nursing Outlook, which was cited 1752 times. The article deals with the effect of exhaustion, the high degree of dissatisfaction with the workplace among nurses, and the organisational support they receive on the quality of healthcare. Another article with a high number of citations (1612) was published in JAMA. In this study, the authors aimed to identify issues related to the quality of healthcare in the United States, including measuring, evaluating, and improving it in healthcare facilities both in the public and private sectors.

Articles published in journals, such as Nursing Outlook, Journal of Service Research, Operations Research, Medical Care, The Lancet, or Environment and Behaviour, have a high number of citations. In addition, articles written by American, Greek, and Dutch authors recorded a high number of citations, while authors from countries such as Jamaica, Qatar, or Romania had a low number of citations.

The evolution over time of the distribution of the mean number of citations can be observed in Figure 3. It can be easily seen that the articles that were most often cited are those published in the first half of the analysed period (between 1975–2013). This result may show that articles that have recorded more citations have a higher intensity of impact on the volume of knowledge. Furthermore, from Figure 3, it can be observed that a high number of articles appeared in the early period of the pandemic. Comparing the number of published articles and the number of citations received, we can believe that research topics from the healthcare management field became more actual and diverse. Regarding this, some inherent risks can be identified. One of the highest risks can be the dissemination of poor-quality research and rapid scientific publication, the quantity of information and processing speed being at an impressive level in this period.

To evaluate the effects of some factors on article citation, a multilevel model was used. Initially, a multilevel model that allows for estimating the journal effects on citation number and with explanatory variables was used. The obtained results are presented in Table 5.

From Table 5, article citation is strongly affected by the time since that article was published. This effect is statistically significant because the estimated coefficient is more than five times its standard error.

Another factor that has a high effect on article citations is the article topic. Our results show that, for topics such as “Impact of different factors on healthcare management” and “Challenges of healthcare management”, the obtained coefficients were higher than the standard error.

However, we have considered only the main effects of these variables, and the relationship between the number of article citations and the explanatory variables may depend on an interaction effect between some explanatory variables. We added those interactions to the model, with estimates presented in Table 6. For the results presented in Table 6, the estimated coefficients for the interaction between variables are higher than their standard errors. However, the addition of those interaction effects does little to explain the differences between journals: the journal-level variance has only increased from 10,643 to 10,752.

To compare the two estimated models, the likelihood ratio test statistics were calculated (Table 7).

From the low Pr (>χ**^2^**) value, we conclude that there is evidence that the second model (Model 2) can more adequately explain the data structure and the effect of interaction between variables, which differs across journals.

## 4. Limitations

The limitation of our study arises from the use of quantitative methods to review the papers published in the healthcare management field. The review relied on the analysis of bibliographic data associated with the documents rather than an examination of the research findings. Thus, the review’s implications are limited to the general directions of the studied field rather than the synthesis of article results. Thus, our results can be used as a starting point for future analyses aimed at the development of this field.

## 5. Discussions

In our study, we aimed to perform a bibliometric analysis of the research literature of articles that treated topics in the healthcare management field. We identified trends in the publication of analysed articles, with an evident global spreading of ideas and into specific topic themes. In addition, we found substantial evidence leading us to make the following statement: a responsible author will publish the results of high-quality research in a way that is easy to understand and attractive, guaranteeing that it will be widely read by several categories of audience and thus accumulating more citations.

Our study delivered additional types of results. Thus, from the results regarding article publication, we saw that the journals in which most of the analysed articles were published are JAMA, Health System in Transition, and Libyan Journal of Medicine.

Regarding the affiliation of the main author, we found that most of the analysed articles are written by researchers from the United States, United Kingdom, Australia, and Canada. This result shows that a high number of analysed articles were published by researchers from developed countries. Similar results were reported for studies in other branches of medicine, such as general medicine [36] and neurology [37]. Regarding the small number of articles published by authors from developing countries, it can be considered that both their quantity and quality may be affected by several barriers, such as poor funding, lack of facilities to support research activities, lack of government incentives, or inadequate support. These issues can contribute to maintaining a scientific environment and research conditions that do not meet the competitive standards of prestigious journals. Certainly, journals can publish research results that are of interest to their readers. However, where possible, journals could support the dissemination of healthcare research conducted in the developing world by setting up international advisory boards to help ensure a greater degree of diversity.

Another result of the study was that before 2000, the only countries that published articles on healthcare management were the United States, United Kingdom, or Germany, and since 2015, articles have come from 19 different countries. This result shows that researchers from different countries have started to be more active since 2000. One possible explanation is that international cooperation in healthcare research between different countries has varied significantly, with the interest in research being gradually spread across multiple aspects of this field. Moreover, after 2000, an increasing trend of scientific production on healthcare management topics can be observed. The geographical distribution of analysed articles reveals that in the last two decades, the research has spread in countries from continents, such as North America and Europe. This result is not surprising, taking into consideration that the developed countries in North America and Europe have a stronger academic research infrastructure in healthcare management than less developed nations since they have more research institutes, well-established data management systems, and more assets allocated for research. The number of authors who are affiliated with research institutions in regions such as Asia, South America, or Africa was lower, and the dissemination of the results of their inquiry manifested later (after 2000).

The interest shown in healthcare management research can, however, also be affected by the healthcare system model specific to each nation or region. Healthcare systems in developed nations (for example, Regional Health Systems (RHS) in Singapore, Managed Care in the United States, or National Health Services (NHS) in the United Kingdom) have implemented several systematised medical lines of action [38,39,40]. The requirement for high-quality healthcare services has elevated many inquiries about health systems. For example, about 10 years ago, China and India deployed health reformation, setting out a set of policies to integrate healthcare services to improve their quality [41]. The objectives of healthcare reformation have made research topics catchier, and research can be more substantially supported and for longer, including through data availability and funding. In addition, the ways for cooperation between academic institutions have also influenced local research productivity [42,43].

Given our results and the results of other studies [44,45], we consider that the degree of economic development of a country or region can influence the production of scientific articles, with our analysis reporting a high growth in scientific production in more developed countries. Regarding this result, we also found evidence of countries with a lower level of GDP per capita for which the scientific production is high (Table 8). This can suggest that there is not a clear link between economic development and scientific production in this field. Instead, this may show an effort made by governments in those countries in terms of investments in healthcare or the interest manifested by their scientific community for this field.

Further, these results suggest that there may be a dilemma between healthcare ethics and financial constraints. In the literature, many empirical studies describing health employees’ dilemmas and decision-making processes can be identified [46,47,48,49]. These studies have, however, focused on developed countries, with a limited number of studies presenting results for the case of low and middle-income countries. Furthermore, the available studies are small qualitative studies [50,51,52,53,54,55].

In our paper, we also aimed to analyse the relationship between the number of articles citations and several factors. Some of those factors were number of authors of an article and the country with which the principal author is affiliated. We assumed that we could identify a correlation between these factors and the number of citations, with a higher number of authors bringing more information as well as personal grid contacts or research infrastructure. Even if authors such as [56,57] reported such effects in their papers, our results showed that there is an effect of author number but not author affiliation. Statistically significant effects were identified regarding the time since an article was published, showing that the temporal dimension is important. Articles that were recently published have not yet been cited, and the number of citations grows with time. In other words, the older an article, the more likely it is to be cited more than once [58].

Another important factor that has a significant effect on the number of articles citations is the title length (number of words in the title). We found that this factor has a negative effect on article citations. Similar results were also found by other researchers [59,60], with their argument being that the shortest titles generate the most citations. However, this effect could depend on differences between fields or research topics. Regarding this, the authors of [61] showed that in medicine, shorter titles often have less citations. On the other hand, the authors of [62] argued that this relationship became inverted around the year 2000 from negative to positive. This situation is explained by the growing volume of online search engines (for example, Web of Knowledge was released in 2002, Scopus in 2004, and Clarivate in 2016). It seems that short titles perform better when the search is made manually, while longer titles produce more hits in online searches, ensuring a higher degree of discoverability and a higher number of citations.

Another important effect on article citations is due to the interaction between the number of pages in an article and the time since it was published. Considered alone, the variable representing the number of pages does not seem to influence the number of citations. Regarding those results, the authors of [63,64,65,66,67,68] showed that the pagination affects the number of article citations depending on the discipline. In general, in the field of medicine, longer articles have more content, and the readers have a greater opportunity to find and access important info, which is conducive to a higher number of citations.

## 6. Conclusions

This paper provides an overview of research tendencies, challenges in healthcare management research, and possible future research perspectives. Healthcare research has grown significantly since 2000. Our results show that there are regional disparities in scientific output that are related to some aspects of economic development, specificities of healthcare systems, and academic association models.

In the paper, we also evaluated the factors influencing the number of citations of the analysed articles published in journals from the healthcare management field. We assume that the significance of identified factors will be sensitive to the growth of other approaches regarding impact quantification.

Thus, our results are useful in providing evidence related to the researched issues. Moreover, they can be used before planning or deciding a strategy for researching various aspects related to healthcare management. Thus, the awareness of the existence of a solid fund of knowledge increases, and the existing gaps can be identified, or some conceptual and methodological inconveniences can be avoided. On the other hand, exploring these data sources can help pave the way for future knowledge management (including science policy), as well as social policy decisions. In addition, our analysis is helpful in identifying unsatisfied challenges in the studied field that can trigger new ideas for further research.

## Figures and Tables

**Table 1:** Other reasons for excluding articles from the analysis.

Reason for Exclusion	Number of Cases
The presented topic does not concern healthcare management	17
The article is not published in an academic journal (the article is published in a non-scientific journal)	20
Information about the article cannot be found	5
Total	42

**Table 2:** Studied variables and their characteristics.

Variables.	Characteristics
Publication year	Numeric
Topic	1 = Concepts and literature review 2 = Healthcare services quality 3 = Access at healthcare services 4 = Healthcare systems 5 = Impact of different factors on healthcare management 6 = Good practices in healthcare management 7 = Healthcare management legislation 8 = Challenges of healthcare management 9 = Other
Journal	Nominal
Article length (page number)	Numeric
Number of authors	Numeric
Number of citations	Numeric
Number of words in the title	Numeric
Number of references	Numeric
Year since publication	Numeric
Country	Nominal

**Table 3:** The first 15 journals in which healthcare management articles have been published.

Journal:	Number of Articles
JAMA	34
Health System in Transition	11
Libyan Journal of Medicine	7
Journal of Healthcare Management	6
Health Services Research	5
Journal of Health and Social Behaviour	5
Medical Care	5
Medical Care Research and Review	4
Waste Management	4
BMC Health Services Research	3
Journal of Cleaner Production	3
Journal of the American Medical Association	3
Rural & Remote Health	3
Social Science & Medicine	3
The Milbank Memorial Fund Quarterly	3

**Table 4:** Number articles and citations according to topic.

Topic:	Number of Articles	Number of Citations
Healthcare management systems and challenges	47	123
Quality of healthcare services	42	100
Healthcare services and access to them	33	79
Concepts and literature review	31	66
Characteristics of health systems regarding healthcare management	31	104
The impact of some factors on healthcare management	30	160
Legislation in the field of healthcare management	20	36
Good practices in healthcare management	16	117
Total	250	-

**Table 5:** The effect of different factors on article citations and a coefficient of random effects according to journal.

Model 1: Fixed Part Parameters:	Estimate	Std. Error	*t*-Value
Intercept	−68.26969	89.08833	−0.766
Words	0.44421	3.16362	0.140
Authors	0.07013	3.79989	0.018
Pages_number	−1.55821	2.38532	−0.653
Year_since_publication	7.54012	1.49733	5.036
Healthcare services quality	77.74738	53.67323	1.449
Access at healthcare services	40.92674	53.06987	0.771
Healthcare systems	28.31368	58.57220	0.483
Impact of different factors on healthcare management	79.42949	52.96303	1.500
Good practices in healthcare management	42.31075	64.17250	0.659
Healthcare management legislation	41.79778	62.19571	0.672
Challenges of healthcare management	120.21847	48.92182	2.457
America	52.32560	69.24696	0.756
Asia	40.31969	74.07866	0.544
Australia	60.28909	87.66612	0.688
Europe	24.05150	70.65020	0.340
Journal-level random effects			
	Variance	Std. Deviation	
$\sigma_{u0}^{2}\;$(Intercept variance)	10,643	103.2	
Individual-level random effects			
$\sigma_{e}^{2}$	32,672	180.8	

**Table 6:** The effect of different factors on article citations with the interaction effect of explanatory variables and a coefficient for random effects according to journal.

Model 2: Fixed Part			
Parameters:	Estimate	Std. Error	*t*-Value
Intercept	35.4375	94.1489	0.376
Authors X Year_since_publication	0.6498	0.4918	1.321
Pages_number X Year_since_publication	−0.4491	0.1804	−2.489
Title_Words X Year_since_publication	1.2164	0.3000	4.055
Title_Words	−7.9166	3.7434	−2.115
Authors	−5.0053	5.4333	−0.921
Pages_number	2.8077	2.9963	0.937
Year_since_publication	−1.4109	4.1719	−0.338
Healthcare services quality	43.7434	52.5286	0.833
Access at healthcare services	−13.0142	52.3086	−0.249
Healthcare systems	−3.5217	56.7524	−0.062
Impact of different factors on healthcare management	44.0956	51.5642	0.855
Good practices in healthcare management	17.7223	62.0884	0.285
Healthcare management legislation	39.0435	59.8099	0.653
Challenges of healthcare management	109.8549	47.0755	2.334
America	33.0154	67.1403	0.492
Asia	24.4895	71.6200	0.342
Australia	52.3341	84.7939	0.617
Europe	14.1147	68.3587	0.206
Journal-level random			
	Variance	Std. Deviation	
$\sigma_{u0}^{2}\;$(Intercept variance)	10,752	103.7	
Individual-level random			
$\sigma_{e}^{2}$	29,292	171.1	

**Table 7:** Likelihood ration test statistics.

	npar	AIC	BIC	logLik	Deviance	Chisq(χ^2^)	Df	Pr(>χ^2^)
Model 1	18	3387	3450.3	−1675.5	3351.0			
Model 2	21	3371.2	3445.1	−1664.6	3329.2	21.733	3	000.000 ***

Signif. codes: 0 ‘***’ 0.001.

**Table 8:** Countries with the highest scientific production in our analysis in relation to GDP per capita and heath expenditure per capita.

Country	Health Expenditure per Capita, 2019 (or Nearest Year) (USD)	GDP per Capita in 2020 (USD)
USA	10,948	63,413.5
UK	4500	41,124.5
Australia	4919	51,692.8
Canada	5370	43,258.2
India	257	1927.7
Germany	6518	46,208.4
China	811	10,434.8
Iran	8662	2422.5
Italy	3653	31,714.2
Libya	6.05	3699.3

Source: World Bank and OECD Health Statistics 2021, WHO Global Health Expenditure Database.

## Data Availability

The data presented in this study are available on request from the corresponding author. The data are not publicly available due to restrictions, e.g., privacy or ethical.
